# Monitoring Movements of Ataxia Patient by Using UWB Technology

**DOI:** 10.3390/s20030931

**Published:** 2020-02-10

**Authors:** Tanjila Akter Zilani, Fadi Al-Turjman, Muhammad Bilal Khan, Nan Zhao, Xiaodong Yang

**Affiliations:** 1 School of Electronic Engineering, Xidian University, Xi’an 710071, China; tanjilazilani@stu.xidian.edu.cn (T.A.Z.); bilal@stu.xidian.edu.cn (M.B.K.); nzhao_3@stu.xidian.edu.cn (N.Z.); 2 Artificial Intelligence Engineering Department, Near East University, 99138 Nicosia, Mersin 10, Turkey; fadi.alturjman@neu.edu.tr; 3 Research Centre for AI and IoT, Near East University, 99138 Nicosia, Mersin 10, Turkey

**Keywords:** UWB, IoMT, Ataxia, SVM

## Abstract

Internet of multimedia things (IoMT) driving innovative product development in health care applications. IoMT requires delay-sensitive and higher bandwidth devices. Ultra-wideband (UWB) technology is a promising solution to improve communication between devices, tracking and monitoring of patients. In the future, this technology has the capability to expand the IoMT world with new capabilities and more devices can be integrated. At the present time, some people face different types of physiological problems because of the damage in different areas of the central nervous system. Thus, they lose their balance coordination. One of these types of coordination problems is named Ataxia, in which patients are unable to control their body movements. This kind of coordination disorder needs a proper supervision system for the caretaker. Previous Ataxia assessment methods are cumbersome and cannot handle regular monitoring and tracking of patients. One of the most challenging tasks is to detect different walking abnormalities of Ataxia patients. In our paper, we present a technique for monitoring and tracking of a patient with the help of UWB technology. This method expands the real-time location systems (RTLS) in the indoor environment by placing wearable receiving tags on the body of Ataxia patients. The location and four different walking movement data are collected by UWB transceiver for the classification and prediction in the two-dimensional path. For accurate classification, we use a support vector machine (SVM) algorithm to clarify the movement variations. Our proposed examined result successfully achieved and the accuracy is above 95%.

## 1. Introduction

The exponential growth of applications in the internet of multimedia things (IoMT) requires higher bandwidth technology especially regular monitoring of patients [1,2]. Ultra-wideband (UWB) technology is one of the solutions for higher bandwidth challenges. It also has continuous tracking and monitoring ability [3]. In this research, UWB technology is used to monitor the Ataxia patients. People who have Ataxia, control movement and balance are affected by their daily behavior. Ataxia has an effect on the fingers, hands, arms, legs, body speech and eye movement. For the diagnosis, patients need to check their movements especially walking movements is important. When they walk, sometimes they feel confused about taking a step. As a result, they are unable to walk with proper balance. Currently, clinically it is difficult to find out the particular problem of the Ataxia patients and also more challenging work to monitoring the movements. The gesture imbalance in Ataxia people used the wearable detector to estimate their position [4]. With the help of UWB technology, detection of human walking activity and human body tracking are possible for actual implementation. UWB technology has greatly improved for human detection and tracking system for accuracy on two-dimensional and three-dimensional paths. For the real-time location (RTL) and wireless sensor networks (WSN) system, UWB technology-based sensor is mainly used as an IEEE 802.15.4-2011 standard. This sensor is a low power radio transceiver, transferring data rate is high and having high bandwidth which is larger than 500 MHz and frequency bands from 3.5 GHz to 6.5 GHz. Recently for safety and security, this technology is used in hospitals, healthcare, supermarket and different industries for tracking and monitoring.

The main purpose of this paper is to monitor the walking movements of Ataxia patients using UWB technology-based for the indoor environment. This system helps to detect the movements by using the UWB sensor and classified using the support vector machine (SVM) algorithm, we classify the collected data to determine every movement. SVM is very famous for data classification and regression analysis [5].

This paper characterizes the variations of the walking movements by using a detection method consisting of a small wireless sensor of UWB technology. We present a system for the detection with monitoring of the abnormal and normal walking activity by the patients suffering from Ataxia. The rest of this paper is coordinated as follows: In Section 2, motivation behind the research work is explained. In Section 3, related work to the proposed research, Section 4 materials and methods are presented. In Section 5, we explain our experimental process, Section 6 gives achieved results and related discussion and finally, Section 7 concludes this work.

## 2. Motivation Work

Recently, UWB technology is the most popular for locating each active element in space precisely. For the short-range UWB radio technology is used in a very low energy level and high-bandwidth communication. Applications of IoMT are now expanding with expeditiously. Monitoring of human activity especially for clinical assessment of the Ataxia patients, people are using IoMT systems [6,7]. Ataxia means a lack of coordination, so it’s quite hard to monitor and find out the estimation of the accurate positions in an indoor environment. It is a new research area for researchers. This encourages researchers for the development of this system [8]. Many technologies and systems are used for monitoring purposes. The major problem of Ataxia is balance disabilities which need regular monitoring and practice physical activity [9]. So, on this point, patient monitoring is the most important for patient’s family members and caretakers. The motivation of the study is to improve the patient’s life suffering from Ataxia with the help of technologies like UWB.

## 3. Related Work

There are many tracking systems used in indoor and outdoor environments. Based on the hardware device, a human movement detection method has become very popular. In Cerebellar Ataxia, for motion measurement, some are used in optoelectronic motion analysis systems [10]. For monitoring of physical activity, some use body-worn or triaxial accelerometer type devices [11]. UWB sensor is constructed with multiple implementation solutions [12]. Detection and tracking of the human body are already recognized by the UWB radar system [13]. With the help of this sensor without radar technology, we can detect human activity. There are various systems are available for the detection of the human body with the wireless sensors. They have different precision positioning systems. UWB RTL system gives the higher precision than others [14]. In most cases for detection and localization, radar technology was used. With dual-frequency in CW radar used for locating moving target for indoor imaging [15], human gait recognition and detection of the localization while people walking is also constructed by UWB radar [16,17]. All the systems are used for location detection but using the UWB sensor, we can also find the variations of the movements. The common symptom in Cerebellar Ataxia is postural balance. In this situation, some used wearable sensors for positioned their locations but UWB technology is more suitable for estimation [18]. Using RFID and harvesting sensors, it is also perceived the body language of humans in All See prototype system [19].

With the comparison of others methods, UWB sensor have transceiver chips which are used for RFID and RTL system application and the chip which measures 6 mm by 6 mm, use a very low energy level to transmit low range and medium-range (about 70 to 250 feet) and short-duration transmission (up to 6.8 Mbps) to gateway receivers. So, UWB based indoor positioning system is very famous for accurate estimation of the location in various movements.

## 4. Materials and Methods

At present, for multimedia application UWB technology gives a different path to wireless technologies compared to very common narrow-band systems, which bring a huge place of interest in the research sector. According to the Federal Communication Commission (FCC) [20], UWB technology bandwidth is larger than 500 MHz and center frequency fc larger than 2.5 GHz. In [21], the signal speed is potentially 10 GHz spectrum and it has low electromagnetic radiations. In an indoor environment, the radio power pulse is lower than −41.3 dB, which has little impact on the environment and low radiations are not harmful to the human body. For the implementation of our method, we use a very simple system which is based on the two-way-ranging (TWR) real-time location systems (RTLS) method in UWB technology. The data rate of this sensor was 6.8 Mbps for short-range and 110 kbps is for long-range. In our experiment, the data rate is 6.8 Mbps. On the other hand, UWB has low processing energy and consumed capacity which is enabled by long-life battery-operated devices. In our method, we use the sensors which compliant with the IEEE 802.15.4-2011 UWB standard. It has a CMOS single-chip radio transceiver integrated circuit (IC). Using TWR in time-of-flight (TOF) it can locate the position with 1-dimension and the detection accuracy is ±10 cm with RTLS, it can detect the location with (2D/3D) and the detection accuracy of ±30 cm using TWR or time-difference-of arrival (TDOA).

**Figure 1:**
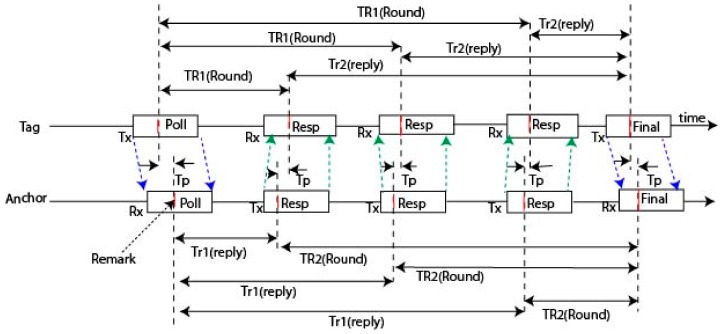
Scenario of the two-way-ranging (TWR) scheme in three anchors with five messages.

There are a number of different methods of implementation for the RTLS system using a wireless device. This device is a simple two-way-ranging-based RTL evaluation system comprised of just three anchors and up to eight tags. TWR is a system where radio transmissions are coordinated by the time between two devices and connected mathematically to determine the distance or range between the devices. With three anchors, the trilateration solver actually gives two solutions equidistant each side of the plane of the anchors, which is assumed to be all horizontal. The anchors are all mounted at the same height above the area of interest for the localization. This device works as a TWR scheme, to estimate the distance between tag and anchor which is shown in Figure 1. Implementation of this method we use a simple TWR based RTLS evaluation system. For the localization with three anchors and one tag. We placed all the anchors at the same height. Instead of performing an individual TWR exchange, the radio message was between the tag and the anchor. This sensor operates an optimized message exchange. In Figure 1, when tag sends a message (poll) and received all anchors, each anchor sends a response message in turn, after that tag completes the ranging exchange by sending a final message and all anchors received the final message. Each device records an accurate transmission and reception timestamp of the message. The tag sends the actual transmission time of the final message, along with the first sending time and response time from each of the anchors. When each anchor received the final message, they record the time stamp between transmission and reception and calculate the time-of-flight (TOF) between the tag and itself. The remark is the part of the frame that is assumed time-stamped at the device antenna.

The final message communicates the tag’s $T_{R}\left(Round\right)\;and\;T_{r}\left(\mathrm{reply}\right)$ times to the anchors and $T_{P}$ is the values of TOF which is the propagation time of the message between tag and anchors. Here, anchor calculates the range of the tag which is given in Equation (1):

(1) $$T_{p}=\frac{T_{R1}\cdot T_{R2}-\;T_{r1}\cdot T_{r2}}{T_{R1}+T_{R2}+T_{r1}+T_{r2}}.$$

The position of the tag can be estimated by TOF from the anchors. For location estimation, it needs to measure the distance between tag and anchors in RTLS. The speed of the radio waves through the air is the same as the speed of light, then the distance of the tag and anchors is:

(2) Distance=C×TOF.

The positioning error may have occurred because of the covered area and error in clock, which is used to represent TOA at each device. All of the features of UWB technology are used for accurate detection and localization, especially for medical monitoring. The Ataxia disease is a hereditary and non-hereditary disease of the cerebellum and spinal cord, where the main symptom is progressive Ataxia. The main symptoms of Ataxia are defined by their cerebellar area damage which occurs during irregular foot placement [22]. We monitor the human leg-coordination defects to detect cerebellar Ataxia. In our work we can find out the combination of postural instability, walking unsteady, lacking leg-placement. For the accuracy of location estimation, UWB can detect the motion of the patient’s foot placements. In this paper, we can detect the abnormality of walking by monitoring on the basis of the problem of Ataxia patients. For accuracy analysis of clinical symptoms, we divided the walking patterns into four types. One of them is a normal walk and others are an unnatural walk. The walking activities are given below:

- Normal walk (NW)
- Difficulty walking in a straight line (DWSL)
- Walking with stomping or heavy step (HS)
- Bending forward while walking (BFWW)

Given this type of problem of Ataxia traditionally described as unsteady, stumbling walking with increased step width. Actually, it is quite difficult to detect pure Ataxia. Because Ataxia occurs form cerebellum injury. As such injury, one of the most characteristic signs of cerebellar damage is walking in Ataxia [23].

Based on walking, we can detect the abnormality of a walk with the comparison of a normal walk. Detection of lacking coordination is based on the tracking number of the footsteps which is taken by the sensor and to record locomotion in two-dimensional space on the log file. The UWB sensor can recognize the variations of movements with high accuracy. For the treatment of those affected by Ataxia, they need coordination exercise [24] and fall-downs are regular symptoms in cerebellar Ataxia [25]. In this case, when they want to walk without any object, they need help. Under these circumstances, our system is very effective for monitoring in the medical center and home for their safety [9].

## 5. Experimental Process

In this section, we describe the experimental set–up, activity performed, data collection and SVM algorithm.

### 5.1. Experimental Setup

For this experiment, we set up our indoor environment into our laboratory corridor at the School of Electronic Engineering, Xidian University. In the implementation of our proposed work, we used UWB wireless evaluation sensors for data collection, a desktop computer, connected with the anchor, for data processing. For this experiment, we considered a 3D space to provide the X-axis Y-axis and *Z*-axis, where the *Z*-axis is the height of the anchor position from the floor. In this experiment, we used three anchors and one tag. Three anchors were placed in a corridor.

**Figure 2:**
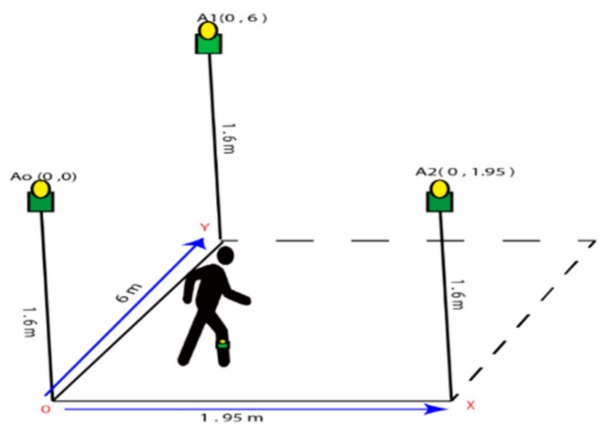
The typical scenario for the experiment.

The length and width of the corridor was 8 m and 3 m respectively. The height of the anchor’s position from the floor was 1.6 m and the measurement of X and Y from the center in an anchor $A_{0}$ to $A_{2}$ were (0, 1.95) m and $A_{0}$ to $A_{1}$ (0, 6) m, respectively. For the mounting of an anchor position, we used hanging stands which can be seen in Figure 2 and the tag can be freely moved to various predefined positions that were fixed on subject’s body in the lower limb portion.

Following the above scenario when the subject is walking within this range the signal can easily detect the different walking activities. All the transaction is recorded into log files between anchors and tag. This device provided the most accurate results. The idle condition of this sensor with 3.993 GHz radio-frequency and the data rate was 110 kbps and the highest location estimation range of the module was 300 m.

### 5.2. Activity Performed

**Figure 3:**
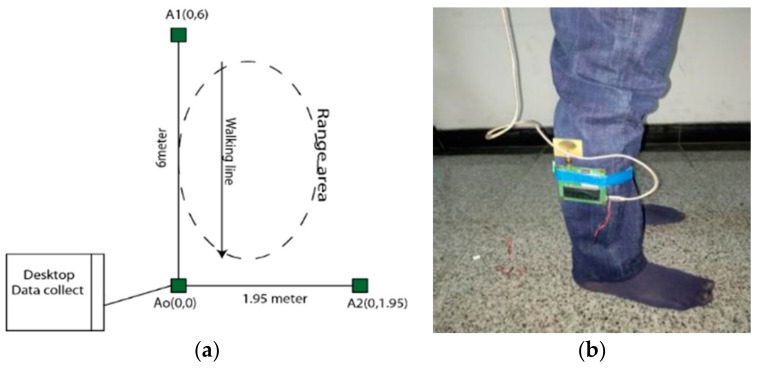
Scenario of the walking task (**a**) the area of walking scenario; (**b**) the sensor used in the experimental object’s body.

According to the Ataxia symptoms, we quantified walking activities into four types and we recorded 2D data using UWB sensors with anchors and tags. When a subject performed in our created scenario, all the data were collected by anchor $A_{o}$ which was connected with the PC as shown in Figure 3. The subject walking within this range. We can compare all the uncoordinated balance walks and normal balance walks, and for this test, it was measured as the forward distance traveled by the lower limb marker while the subject walked. During the subject walking period, we observed the moving tag on the screen and the different movements looked different from one symptom to another.

**Figure 4:**
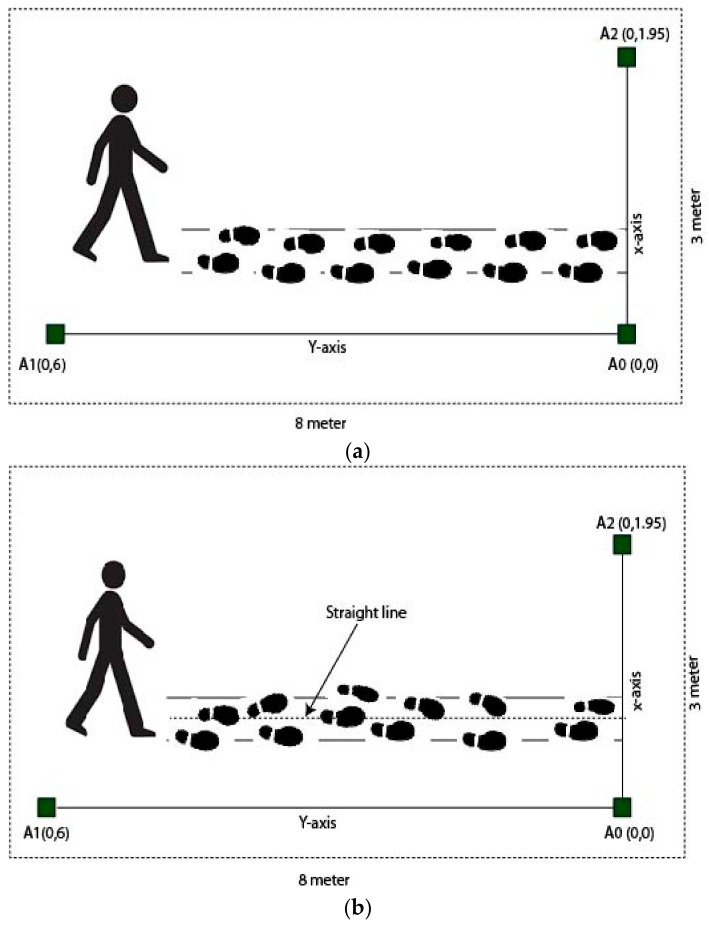

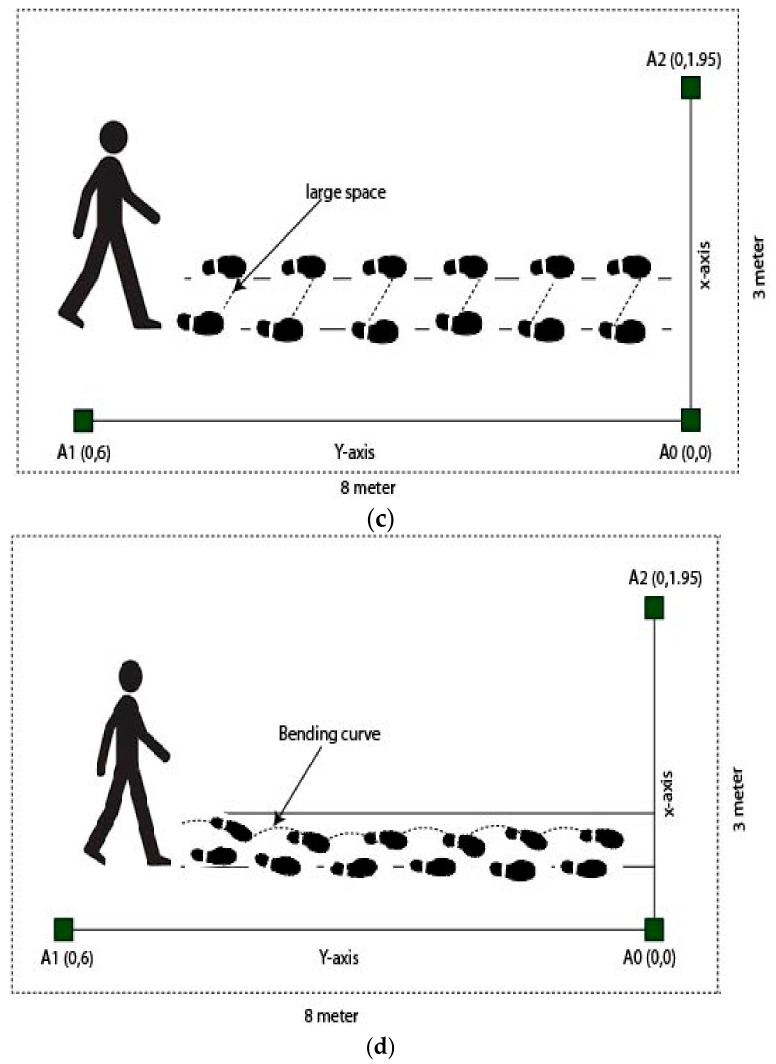
The scenario of the preconstructed walking pattern, (**a**) normal walk (NW), (**b**) difficulty walking in a straight line (DWSL), (**c**) walking with stomping or heavy step (HS), and (**d**) bending forward while walking (BFWW).

With our method, we define four types of symptoms which were preconstructed and all the subjects walked on this pattern. For data classification, the changing position of the tag mounted on the leg due to different footpath gives data for each type of symptoms, where higher data values indicated increased curvature of the footpath. The typical scenario of preconstructed walking movement pattern is shown in Figure 4.

### 5.3. Data Collection

**Figure 5:**
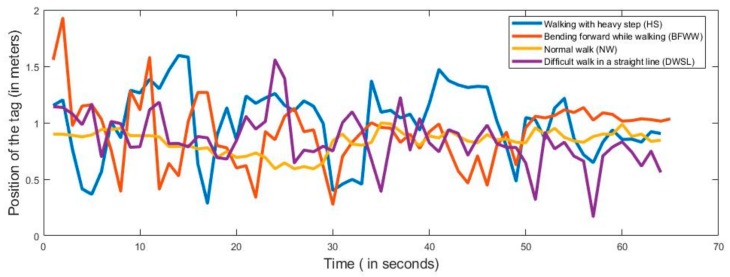
Measured horizontal values for normal and abnormal movements.

**Figure 6:**
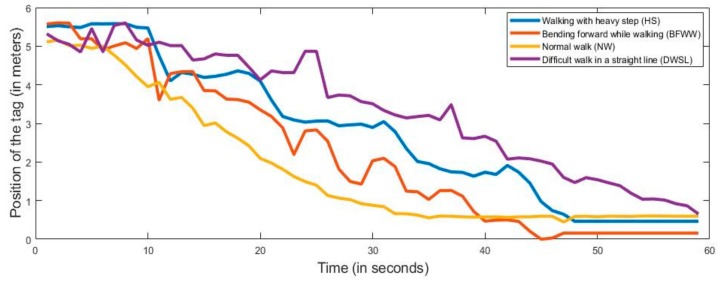
Measured vertical values for normal and abnormal movements.

On the basis of our proposed method, we monitored the detection of walking abnormality. Our experimental task divided four parts on the basis of symptoms such as NW, DWSL, BFWW, and HS. When the subjects performed the walking test, the anchors and tags continuously distributed their signals. For the experiments, nine persons participated, and their ages ranged from 26 to 35 years. They performed 10 times for each symptom and by using the UWB RTL system, we collect the data. We captured the data for more than 60 s, but for classifying we took 60 s in each datum. Therefore, we collected the data in total 360 (symptoms of four types, 10 performances, and nine subjects). After all these experiments, we also capture the activities data into the log file from PC where the software (RTLS-PC) consists of a log file. Every location was estimated by the value of X, Y and Z in the log file. We only took the X and Y values because our experimental sensor device is a two-dimensional tracking device and Z is the height of the anchor position from the floor which is the same for all anchors. In Figure 5, we present the horizontal movements of the subject where the y-axis is the position of the tag and the x-axis presents the time. In Figure 6, we present the vertical movements of the subject, where the y-axis presents the position of the tag and the x-axis presents the time. We use our sensor (tag) on the patient’s lower limb portion of the leg and all of the data we take is the position of the tag in every activity.

According to Figure 5 and Figure 6, we can see the divergence of all the activities. Here, all the movements are different from each other because of their different behavior and the time of the performance is also different. We also examined the performance time of all the movements and their variations. With the comparison of every activity from Figure 5 and Figure 6, it looks as though every movement of the subject is not the same. These results are considered for the classification of normal and abnormal movements using a machine learning algorithm.

### 5.4. Support Vector Machine (SVM)

For the accuracy test, the SVM algorithm was applied on the extracted data. For data separation and for the high dimensional space, the two separate parallel hyperplanes were constructed for maximal separating hyperplanes on each side in SVM [26]. Due to its capability and efficacy, the SVM was very convenient for accessing a large amount of data classification [27]. The error of experimental classification is minimizing and maximizing geometric margin it is called the maximum margin classifier [28]. For this experiment, we use the number of the performance of data and classified the variations of each performance using SVM. For the data training, we expected the number of data to be ($a_{i},b_{j}),$ where i=1,2,3,……z, where z is the number of the experiments, and all the experiments are leveled by support vectors which are separate from the highest margin hyperplanes. It depended on training data in SVM with the help of parameter for separable parallel hyperplane construction and is given by:

(3) S·a+t=0,

where S is for the dimensional real vector and t is for a scalar. Separable hyperplane S point perpendicular and t support to expand the margin. The maximum margin particularly placed on the SVM and the parallel hyperplanes are constructed by,

(4) S·a+t=±1.

Both hyperplanes can select for the linearly separable training data, therefore, there are no marks to expand their distance and the distance between hyperplanes is $2/\left|S\right|$. Experimental data are called support vectors (SVs) which are included with the hyperplanes. The separable hyperplane with the highest margin can be defined by $M_{H}=2/\left|S\right|$. This margin is used to provide SVs means of training data which is denoted by,

(5) $$b_{i}\left[S^{T}\cdot a_{i}+t\right]=1.$$

The optimal separating hyperplane (OCH) contain a maximum margin which is $\left\|S^{2}\right\|$ used to minimize margin, denoted by

(6) $$b_{i}\left[S^{T}\cdot a_{i}+t\right]\geq 1;$$

where i=1,2,3…..z and $a_{i}$ is a training vector. After that, when the saddle points of the Lagrange’s solved the optimization problem then there are many functions found [5,29]. The kernel function, $K\left(a_{i},a_{j}\right)\equiv \Phi {\left(a_{i}\right)}^{T}\Phi \left(a_{j}\right)$ is established with a larger margin separable hyperplane and it performs in high dimensional which is very famous. In SVM there are many kernel functions are used. The primary kernel functions are linear, radial basis function (RBF) kernel and sigmoid kernel. For classification of the walking movements in this experiment, we used sigmoid and RBF kernel function to calculate the distance by using Equations (7) and (8) respectively:

A sigmoid kernel function:

(7) $$K\left(a_{i},\;a_{j}\right)=\tan h(\gamma a_{i}{}^{T}a_{j}+r).$$

**Figure 7:**
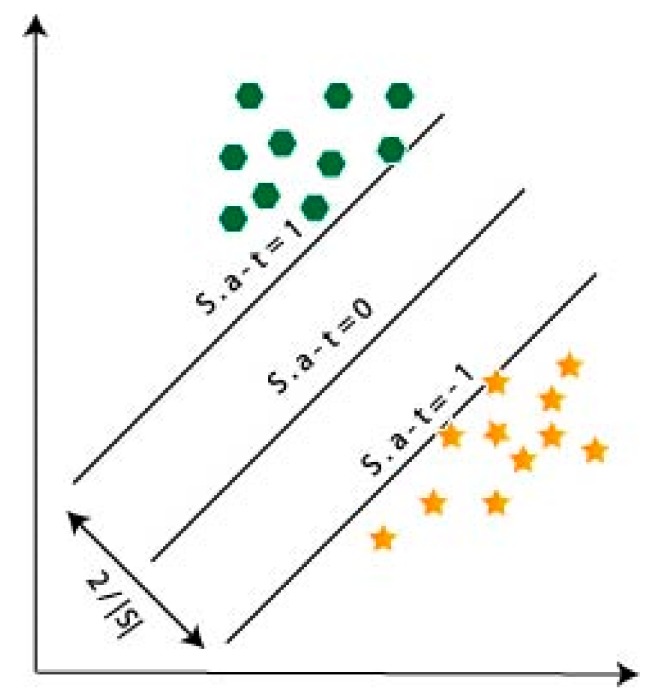
Typical outline of the maximum margin hyperplanes in two classes of support vector machine (SVM).

A radial basis kernel function:

(8) $$K\left(a_{i},a_{j}\right)=\exp\left(-\gamma \|a_{i}-a_{j}\|{}^{2}\right),\;\gamma >0,$$

where r and γ are the kernel parameters. According to two functions, we trained our data for the classification. In Figure 7, we present a classification outline with SVM for two classes. If we use more than two classes, then two approaches, one-against-one and one-against-all, were used in the experiment are defined in [30,31].

## 6. Results and Discussion

In this section, we present the results of the experiment and classifying extracted data by using the SVM algorithm. In our experiment, we used two SVM functions to classify our collected data. We classify the vertical and horizontal data altogether (720 data). In our experiment, we performed classification based on the different walking activities performed. We separated the dataset into testing and training proportion and each proportion behaves as a section which is shown below:

Considering Table 1 for all the activities, we used SVM to classify the data to find the abnormality of the walk.

### 6.1. Experimental Result

**Figure 8:**
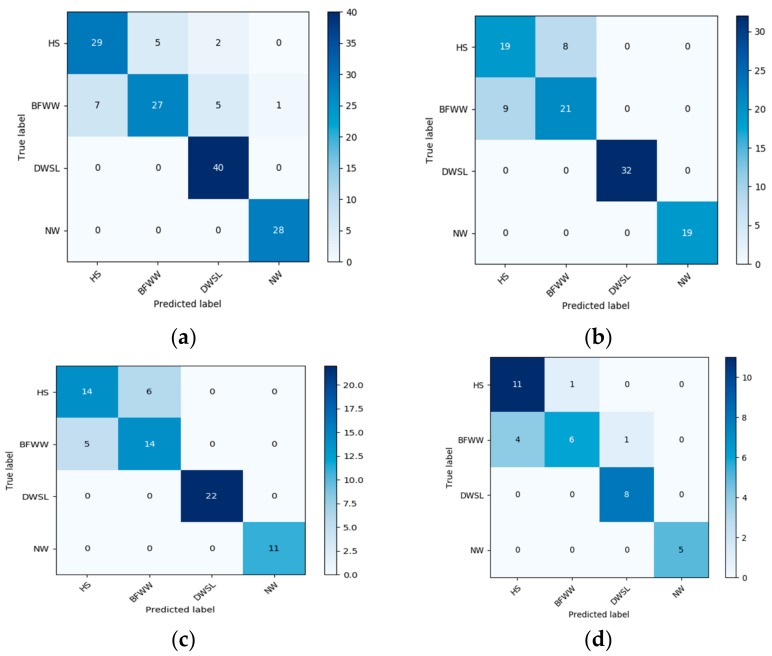
The result of the confusion matrix of four activity using SVM-sigmoid kernel function (**a**) 60% training and 40% testing; (**b**) 70% training and 30% testing; (**c**) 80% training and 20% testing and (**d**) 90% testing and 10% training.

**Figure 9:**
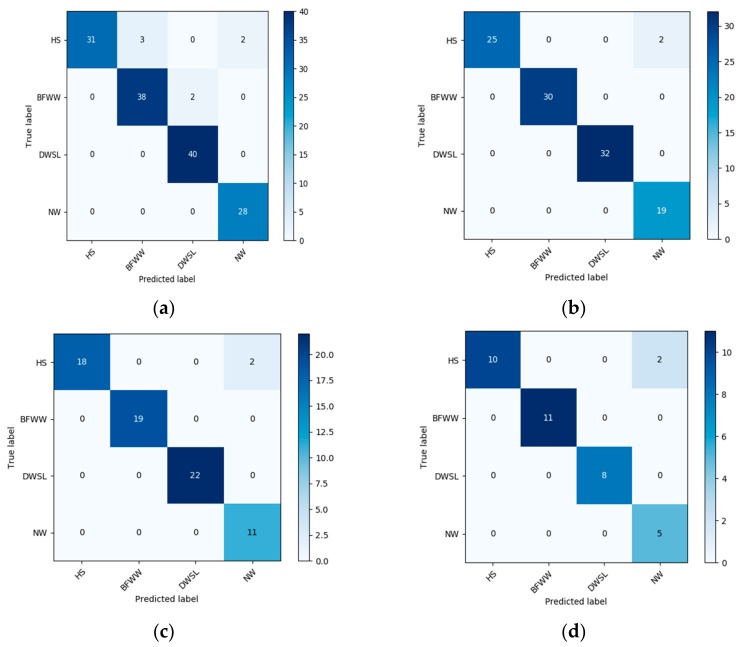
The result of the confusion matrix of four activity using SVM-radial basis function (RBF) kernel function (**a**) 60% training and 40% testing; (**b**) 70% training and 30% testing; (**c**) 80% training and 20% testing and (**d**) 90% testing and 10% training.

This section explains the experimental result of the trained data by using SVM. We use RBFs and sigmoid function as kernel functions of SVM in the time of classification. The data classification result presented a confusion matrix that shows the accuracy result of a different activity. The confusion matrix of every group for both the SVM algorithm presented in Figure 8 and Figure 9. The confusion matrix of the sigmoid kernel function is given below:

According to Figure 8, we make a predictable Table 2 for showing the result of each activity in every section which is given below:

Here on the basis of this table, it is showed that every monitoring activity is different from each other. Only in section (A), the performing result of BFWW is different from other sections and DWSL is different in every section and other activities result in approximately the same.

The confusion matrix helps us to understand how the accuracy result came out and which class miss predict with which class. The confusion matrix of the radial basis kernel function is given below.

For the clear concept of the result of the RBF kernel function we also use a predictable Table 3 to show every section data training and testing results.

According to Table 3, we also see the differentiation of each activity. From all the information in the above table we can see the accuracy result of training and testing data set, which has very good accuracy. The different values of this function are only for a normal walk and the results of other activities in every section are the same.

Compared with the sigmoid kernel function when we use the RBF kernel function for all the unnatural and normal walk, the percentage of data accuracy increased rapidly. This was an increase in testing samples, the highest accuracy achieved using the RBF kernel function while all the misstep data reduced.

### 6.2. Discussion

After all these classification results, we discuss the accuracy of the results of all the data. UWB sensor is observed in a particular signal for these activities that are clearly distinguishable. The SVM train function is used to classify the activities and representing the abnormality walk. In SVM the two kernel functions sigmoid kernel and RBF were used. Analyzing the results by using sigmoid kernel function the data accuracy rate is 86.11%, 84.26%, 84.72%, and 83.34% respectively for HS, BFWW, DWSL, and NW walk.

In Table 4, we see the result of all the performances in every section with two SVM kernel functions. So comparatively using the RBF kernel function in SVM, the result is higher than the sigmoid function. Simultaneously, the accuracy rate of the data is 95.14%, 98.15%, 97.22% and 94.44% for the four types of walk. So, after observation of this confusion matrix of all data using a two-kernel functions for the following four groups, we can determine that all levels are different from each other, and the similarity rate between all levels is low and also depends on the activity performed.

On the other hand, we make a predicted level with the value of positive and negative rates at every performance. We conduct accuracy, true positive rate (TPR present in $R_{P}$ and $R_{N}$) false-negative rate (FNR present in $L_{N}$ and $L_{p}$), and precision, recall and D-measure performance metrics. All the performance data we calculated by:

(9) $$Accuracy=\frac{R_{P}+R_{N}}{R_{P}+L_{P}+R_{N}+L_{N}}$$

(10) $$Precision=\frac{R_{P}}{R_{P}+L_{P}}$$

(11) $$Recall=\frac{R_{P}}{R_{P}+L_{N}}$$

(12) $$D-measure=\frac{2*Precision*Recall}{Precison+Recall}.$$

All the information related to true positive and false negative values is given in Table 5.

According to the above discussion, the performing result of the sigmoid kernel function is lower than the RBF kernel function for all the data. SVM using RBF gives a better accuracy rate. So, this result showed very good accuracy in an indoor environment with estimating an accurate shape that provides a baseline for measurements. At present time, there are several sensor devices that are produced to use in IoMT but we focused on UWB technology because of its accuracy and higher bandwidth.

This paper focused on an indoor environment positioning system for a single person in a specific direction and the result is authenticity. In the near future, this system can be done for the monitoring of multiple persons and outdoor positioning systems. This system can be exploited in several healthcare applications with charms of UWB technology higher bandwidth and reliability features [32,33,34].

## 7. Conclusions

The IoMT can be used in medical applications that can connect to healthcare information technology systems using networking technologies. By using this system, patients are connected to their physicians and allowing the transfer of medical data over a secure network. The internet estimates the accurate position; it is necessary to have location-based information in order to give a significant health condition information. In our paper, we present a method for monitoring of the Ataxia patients by tracking movements in an indoor environment. We used UWB technology for detection of the walking variations on the basis of walking disabilities in Ataxia patients which will be very harmful if it is not monitored regularly. The proposed method has been implemented by the detection of the particular variations of the walking movements with time. The accuracy of the results showed that our method is very useful for the tracking system. The results achieved by using SVM algorithms for normal and abnormal walking is above 95%. Comparatively, with high accuracy and good performance, this method provides a system for monitoring that can be very helpful in IoMT applications.

## Figures and Tables

**Table 1:** Separate the data set in different proportions.

Proportion	Section
60% of training and 40% testing	A
70% of training and 30% testing	B
80% of training and 20% testing	C
90% of training and 10% testing	D

**Table 2:** The predicted level of four symptoms with true level, training and testing data set in every section in SVM-sigmoid kernel function.

Symptom True Level	Predicted Level in Sigmoid Function Result of 60% Training and 40% Testing Data (Section A)			
	HS	BFWW	DWSL	NW
Heavy step (HS)	29	5	2	0
Bending forward while walking (BFWW)	7	27	5	1
Difficulty walking in straight line (DWSL)	0	0	40	0
Normal walk (NW)	0	0	0	28
	**Result of 70% Training and 30% Testing Data (Section B)**			
	**HS**	**BFWW**	**DWSL**	**NW**
Heavy step (HS)	19	8	0	0
Bending forward while walking (BFWW)	9	21	0	0
Difficulty walking in straight line (DWSL)	0	0	32	0
Normal walk (NW)	0	0	0	19
	**Result of 80% Training and 20% Testing Data (Section C)**			
	**HS**	**BFWW**	**DWSL**	**NW**
Heavy step (HS)	14	6	0	0
Bending forward while walking (BFWW)	5	14	0	0
Difficulty walking in straight line (DWSL)	0	0	22	0
Normal walk (NW)	0	0	0	11
	**Result of 90% Training and 10% Testing Data (Section D)**			
	**HS**	**BFWW**	**DWSL**	**NW**
Heavy step (HS)	11	1	0	0
Bending forward while walking (BFWW)	4	6	1	0
Difficulty walking in straight line (DWSL)	0	0	8	0
Normal walk (NW)	0	0	0	5

**Table 3:** The predicted level of four symptoms with true level, training and testing data set in every section in SVM-RBF kernel function.

True Level	Result of 60% Training and 40% Testing Data (Section A)			
	HS	BFWW	DWSL	NW
Heavy step (HS)	31	3	0	2
Bending forward while walking (BFWW)	0	38	2	0
Difficulty walking in straight line (DWSL)	0	0	40	0
Normal walk (NW)	0	0	0	28
	**Result of 70% Training and 30% Testing Data (Section B)**			
	**HS**	**BFWW**	**DWSL**	**NW**
Heavy step (HS)	25	0	0	2
Bending forward while walking (BFWW)	0	30	0	0
Difficulty walking in straight line (DWSL)	0	0	32	0
Normal walk (NW)	0	0	0	19
	**Result of 80% Training and 20% Testing Data (Section C)**			
	**HS**	**BFWW**	**DWSL**	**NW**
Heavy step (HS)	18	0	0	2
Bending forward while walking (BFWW)	0	19	0	0
Difficulty walking in straight line (DWSL)	0	0	22	0
Normal walk (NW)	0	0	0	11
	**Result of 90% Training and 10% Testing Data (Section D)**			
	**HS**	**BFWW**	**DWSL**	**NW**
Heavy step (HS)	10	0	0	2
Bending forward while walking (BFWW)	0	11	0	0
Difficulty walking in straight line (DWSL)	0	0	8	0
Normal walk (NW)	0	0	0	5

**Table 4:** Results of the data accuracy in the Sigmoid kernel and RBF kernel function in SVM.

Activity	4 Posture of Walk	Sigmoid SVM Classification Accuracy (%)	RBF SVM Classification Accuracy (%)
A	Walking with heavy step (HS)	86.11%	95.14%
B	Bending forward while walking (BFWW)	84.26%	98.15%
C	Difficulty walking in a straight line (DWSL)	84.72%	97.22%
D	Normal walk (NW)	83.34%	94.44%

**Table 5:** Results of the true positive and false negative rate using SVM.

	Sigmoid SVM		RBF SVM	
4 Posture of Walk	True Positive Rate (%)	False-Negative Rate (%)	True Positive Rate (%)	False-Negative Rate (%)
Walking with heavy step (HS)	67	33	97	3
Bending forward while walking (BFWW)	90	10	93	7
Difficult waking in a straight line (DWSL)	100	0	100	0
Normal walk (NW)	100	0	100	0

## References

[1] Renuka K., Kumar S., Kumari S., Chen C.-M. (2019). Cryptanalysis and Improvement of a Privacy-Preserving Three-Factor Authentication Protocol for Wireless Sensor Networks. Sensors.

[2] Alvi S.A., Afzal B., Shah G.A., Atzori L., Mahmood W. (2015). Internet of Multimedia Things: Vision and Challenges. Ad Hoc Netw..

[3] Huang X., Wang F., Zhang J., Hu Z., Jin J. (2019). A Posture Recognition Method Based on Indoor Positioning Technology. Sensors.

[4] Nguyen N., Phan D., Pathirana P.N., Horne M., Power L., Szmulewicz D. (2018). Quantification of Axial Abnormality Due to Cerebellar Ataxia with Inertial Measurements. Sensors.

[5] Vapnik V.N. (1999). An overview of statistical learning theory. IEEE Trans. Neural Netw..

[6] Khernane N., Couchot J.-F., Mostefaoui A. (2018). Maximum Network Lifetime with Optimal Power/Rate and Routing trade-off for Wireless Multimedia sensor Networks. Comput. Commun..

[7] Clausi S., Aloise F., Contento M.P., Pizzamiglio L., Molinari M., Leggio M. (2014). Monitoring mood states in everyday life: A new device for patients with cerebellar ataxia. Psychiatry Res..

[8] Liu H., Darabi H., Banerjee P., Liu J. (2007). Survey of Wireless Indoor Positioning Techniques and Systems. IEEE Trans. Syst. Man Cybern. Part C Appl. Rev..

[9] Marquer A., Barbieri G., Pérennou D. (2014). The assessment and treatment of postural disorders in cerebellar ataxia: A systematic review. Ann. Phys. Rehabil. Med..

[10] Conte C., Pierelli F., Casali C., Ranavolo A., Draicchio F., Martino G., Harfoush M., Padua L., Coppola G., Sandrini G. (2014). Upper Body Kinematics in Patients with Cerebellar Ataxia. Cerebellum.

[11] Giggins O.M., Clay I., Walsh L. (2017). Physical Activity Monitoring in Patients with Neurological Disorders: A Review of Novel Body-Worn Devices. Digit. Biomark..

[12] (2017). Decawave TREK1000 Indoor Localization Solution. http://www.decawave.com/products/trek1000.

[13] Dai X., Zhou Z., Zhang J., Davidson B. Ultra-wideband radar-based human body landmark detection and tracking with biomedical constraints for human motion measuring. Proceedings of the 2014 48th Asilomar Conference on Signals, Systems and Computers.

[14] Kai Pin Tan D., Lesturgie M., Sun H., Lu Y. Moving Target Localization Using Dual-Frequency CW Radar for Urban Sensing Applications. Proceedings of the 2009 International Radar Conference “Surveillance for a Safer World” (RADAR 2009).

[15] Ram S.S., Li Y., Lin A., Ling H. (2008). Doppler-based detection and tracking of humans in indoor environments. J. Frankl. Inst..

[16] Chen V., Li F., Ho S.S., Wechsler H. (2003). Analysis of Micro-Doppler Signatures. IEE Proc. Radar Sonar Navig..

[17] Bharadwaj R., Swaisaenyakorn S., Parini C.G., Batchelor J., Alomainy A. (2014). Localization of Wearable Ultrawideband Antennas for Motion Capture Applications. IEEE Antennas Wirel. Propag. Lett..

[18] Yu K., Oppermann I. Performance of UWB Position Estimation Based on Time-of-Arrival Measurements. Proceedings of the 2004 International Workshop on Ultra Wideband Systems Joint with Conference on Ultra Wideband Systems and Technologies.

[19] Kellogg B., Talla V., Gollakota S. Bringing gesture recognition to all devices. Proceedings of the 11th USENIX Conference on Networked Systems Design and Implementation (NSDI’14).

[20] Gatouillat A., Badr Y., Massot B., Sejdić E. (2018). Internet of Medical Things: A Review of Recent Contributions Dealing with Cyber-Physical Systems in Medicine. IEEE Internet Things J..

[21] Rahayu Y., Rahman T.A., Ngah R., Hall P.S. Ultra wideband technology and its applications. Proceedings of the 2008 5th IFIP International Conference on Wireless and Optical Communications Networks.

[22] Mitoma H., Manto M. (2016). The physiological basis of therapies for cerebellar ataxias. Ther. Adv. Neurol. Disord..

[23] Ashizawa T., Xia G. (2016). Ataxia. Contin. Lifelong Learn. Neurol..

[24] Keller J.L., Bastian A.J. (2014). A home balance exercise program improves walking in people with cerebellar ataxia. Neurorehabilit. Neural Repair.

[25] Schniepp R., Wühr M., Schlick C., Huth S., Pradhan C., Dieterich M., Brandt T., Jahn K. (2014). Increased gait variability is associated with the history of falls in patients with cerebellar ataxia. J. Neurol..

[26] Prabhu P., Karunakar A., Anitha H., Pradhan N. (2018). Classification of gait signals into different neurodegenerative diseases using statistical analysis and recurrence quantification analysis. Pattern Recognit. Lett..

[27] Awad M., Khanna R. (2015). Efficient Learning Machines: Theories, Concepts, and Applications for Engineers and System Designers.

[28] Afif M.H., Hedar A.-R. Data classification using support vector machine integrated with scatter search method. Proceedings of the 2012 Japan-Egypt Conference on Electronics, Communications and Computers.

[29] Boser B.E., Guyon I.M., Vapnik V.N. A training algorithm for optimal margin classifiers. Proceedings of the Fifth Annual Workshop on Computational Learning Theory.

[30] Kumar V., Garg M.L. (2018). Predictive Analytics: A Review of Trends and Techniques. Int. J. Comput. Appl..

[31] Xiong T., Cherkassky V. A combined SVM and LDA approach for classification. Proceedings of the 2005 IEEE International Joint Conference on Neural Networks.

[32] Deebak B.D., Al-Turjman F., Aloqaily M., Alfandi O. (2019). An Authentic-Based Privacy Preservation Protocol for Smart e-Healthcare Systems in IoT. IEEE Access.

[33] Al-Turjman F., Nawaz M.H., Ulusar U.D. (2020). Intelligence in the Internet of Medical Things Era: A Systematic Review of Current and Future Trends. Comput. Commun..

[34] Al-Turjman F., Zahmatkesh H., Mostarda L. (2019). Quantifying Uncertainty in Internet of Medical Things and Big-Data Services Using Intelligence and Deep Learning. IEEE Access.

